# Nanoscale Copper–Tin Dioxide Interfaces for Efficient CO_2_ Electroreduction to Formic Acid and Formate at High Rates

**DOI:** 10.1002/cssc.202501686

**Published:** 2025-11-03

**Authors:** Lan Huang, Felicia Di Costola, Marco Allione, Stefano Bianco, Adriano Sacco, Candido F. Pirri, Juqin Zeng

**Affiliations:** ^1^ Centre for Sustainable Future Technologies (CSFT) Istituto Italiano di Tecnologia – IIT Via Livorno 60 10144 Turin Italy; ^2^ Department of Applied Science and Technology (DISAT) Politecnico di Torino Corso Duca degli Abruzzi 24 10129 Turin Italy

**Keywords:** bimetallic electrocatalysts, carbon dioxide conversion, formic acid, microwave‐assisted synthesis, selectivity

## Abstract

This study reports highly selective and stable electrocatalysts consisting of Cu and Sn for efficient reduction of CO_2_ to formate (HCOO^−^) and formic acid (HCOOH). The copper–tin dioxide catalyst (CuSn) is prepared through a one‐pot microwave‐assisted solvothermal method. Different samples are obtained by varying Cu‐to‐Sn atomic ratios from 4 to 1. When Sn content is low, the CuSn sample shows a main crystalline phase of cuprous oxide (Cu_2_O), with low amounts of surface CuO and SnO_2_ species. An increase in Sn percentage leads to the appearance of the crystalline phase of metallic Cu and SnO_2_, which becomes dominant for the sample with the highest Sn content. During CO_2_ electrolysis, the optimal CuSn material shows a faradaic efficiency (FE) for HCOO^−^ greater than 90% at −200 mA cm^−2^ in a flow cell with alkaline electrolyte. It also performs well under acidic condition (pH 3) to produce HCOOH with an FE of 70% at the same current density. A long‐term test is carried out in 1 M KOH at −100 mA cm^−2^ for over 20 h, showing good retention in FE_HCOO−_. This study highlights the potential of implementing low‐cost catalysts for CO_2_ conversion at an industrial scale.

## Graphical abstract

This work highlights the green synthesis of Cu–Sn nanocomposites for the efficient electrochemical reduction of CO_2_ to formate and formic acid. It demonstrates a sustainable pathway for developing high‐performance, low‐cost, and noncritical raw‐material catalysts for industrial‐scale CO_2_ conversion into valuable chemical products.

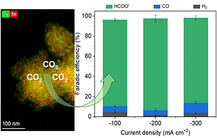

## 1 Introduction

In recent decades, the overexploitation and intense use of fossil fuels have led to rapid depletion of fuel availability and increased CO_2_ emissions. Electrochemical conversion of CO_2_ to produce high‐value fuels and chemicals is a promising pathway to mitigate the CO_2_‐related greenhouse effect and promote economic sustainability.^[^ ^1^, ^2^, ^3^, ^4^, ^5^, ^6^ ^]^ Among many products, formic acid (HCOOH) is of high interest as a convenient hydrogen storage medium via reversible CO_2_ hydrogenation.^[^ ^7^, ^8^ ^]^

At present, various metals such as tin (Sn),^[^ ^9^, ^10^ ^]^ indium (In),^[^ ^11^ ^]^ bismuth (Bi),^[^ ^12^, ^13^ ^]^ cobalt (Co),^[^ ^14^ ^]^ and palladium (Pd)^[^ ^15^ ^]^ have been investigated as catalysts to produce HCOOH or formate (HCOO^−^) from electrochemical CO_2_ reduction reaction (CO_2_RR). Among these metals, Sn electrocatalysts have attracted particular attention since it is a noncritically raw metal and have exhibited excellent selectivity for CO_2_‐to‐HCOO^−^ (or HCOOH) conversion.^[^ ^16^, ^17^ ^]^ However, industrial deployment remains limited by the high overpotentials required to sustain high current densities with good faradaic efficiencies, as well as by the low stability of Sn‐based catalysts.^[^ ^18^ ^]^ Alloying Sn with other metals could be a promising strategy. It is well known that Cu is effective in activating CO_2_ molecule and uniquely converts it into hydrocarbon products,^[^ ^19^, ^20^, ^21^, ^22^ ^]^ while its selectivity for HCOO**^**−**^** or HCOOH is very limited. Combining Sn with Cu could take advantage of both Sn and Cu, maintaining excellent selectivity for HCOO**^**−**^** (or HCOOH) at relatively low overpotentials and improved stability. Some works have been reported to deliver an excellent CO_2_‐to‐HCOO^−^ conversion by tuning the composition or structure of the CuSn bimetallic catalysts.^[^ ^23^, ^24^, ^25^, ^26^, ^27^, ^28^, ^29^ ^]^ Wang et al.^[^ ^27^ ^]^ reported that the excellent activity in electrocatalytic reduction of CO_2_ to HCOO^−^ is ascribed to the abundant Cu/SnO_2_ interfaces in the heterostructure, by reducing the reaction free energies for the formation of HCOO^−^ species. Lei et al.^[^ ^28^ ^]^ achieved effective performance for the electrochemical CO_2_ reduction to HCOO^−^ through reconstruction of CuS/SnO_2_‐S nanoparticles to Cu/Sn/Cu_6.26_Sn_5_ nanowires. Gunji et al.^[^ ^29^ ^]^ reported that carbon‐black‐supported Cu_6_Sn_5_ core–shell nanoparticles give 65% faradaic efficiency for HCOO^−^ at ≈−0.6 V, superior to Cu or Sn alone, highlighting the importance of intermetallic‐core/oxide‐shell synergy. However, most of the previously reported electrocatalysts still have limitations for HCOO^−^ generation via CO_2_RR, such as complicated preparation methods and limited working conditions such as low current densities and narrow potential windows.

In this regard, we present a facile one‐pot microwave‐assisted method for the synthesis of CuSn‐based nanocomposites for efficient CO_2_RR. The optimal CuSn material exhibits excellent selectivity and good stability for HCOO^−^ and HCOOH production across a wide range of current densities in alkaline and acidic electrolytes, respectively.

## 2 Experimental Section

### 2.1 Chemicals

Copper (II) acetate hydrate [Cu (OAc)_2_ · xH_2_O], tin sulfate (SnSO_4_), ethylene glycol, potassium hydroxide (KOH), and potassium sulfate (K_2_SO_4_) of analytical grade were purchased from Merck. All chemicals were used as received. Deionized water was supplied by MyMilli‐Q Lab Water Systems (18.2 MΩ cm, 25 °C).

### 2.2 Synthesis

**Scheme 1:**
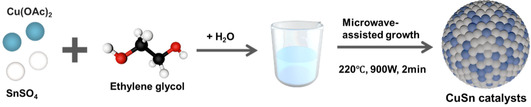
Illustration of the synthesis process of CuSn catalysts.

The preparation of CuSn bimetallic catalyst is schematically represented in **Scheme** **1**, through a microwave‐assisted process. Initially, 40 mL ethylene glycol (EG) and 5 mL H_2_O were mixed. 2.5 mmol Cu (OAc)_2_ · xH_2_O and 2.5 mmol SnSO_4_ were added to the mixture and stirred for 1 h until the complete dissolution of the salts. The solution was then transferred into a Teflon vessel and irradiated for 2 min (*T*_max_ = 220 °C, Power_max_ = 900 W). After cooling down to 60 °C, the precipitates were washed twice with water and once with ethanol, each time centrifuged at 4900 rpm for 10 min, and then dried under vacuum. The obtained catalyst prepared was named Cu_5_Sn_5_. The nanocomposites with different atomic ratios of Cu‐to‐Sn were prepared by changing the ratio of Cu and Sn precursors (8:2 and 7:3), and the total mole of the metal salts was kept at 5 mmol for each synthesis. The synthesized catalyst with only Cu (OAc)_2_ · xH_2_O precursor was denoted as Cu_2_O catalyst.

### 2.3 Physicochemical Characterization

X‐ray diffraction (XRD) was performed in Bragg–Brentano symmetric geometry by using a PANalytical X’Pert Pro instrument (CuKa radiation, 40 kV and 30 mA) equipped with an X’Celerator detector.

Field emission scanning electron microscopy (FESEM, ZEISS Auriga) was used to evaluate the morphology of the materials. For transmission electron microscopy (TEM) analysis, the sample was deposited on a commercial lacey carbon film suspended on an Au grid (Ted Pella Inc., USA) and analyzed in a Talos F200X TEM operated at its maximum acceleration voltage of 200 kV, equipped with an in‐column energy‐dispersive X‐ray (EDX) detector for elemental analysis.

The actual atomic ratios of Cu to Sn in the CuSn samples were determined by inductively coupled plasma–optical emission spectrometry (ICP–OES) analysis.

Raman spectra were acquired on solid powders deposited on a microscope slide by means of a Renishaw InVia Reflex micro‐Raman spectrometer, equipped with a cooled Charge Coupled Device (CCD) camera. The light source was a laser diode (*λ*ex = 514.5 nm) with power lower than 10 mW, and sample inspection occurred through a microscope objective (50×), in backscattering light collection conditions.

### 2.4 Preparation of Electrodes

Herein, a drop‐casting method was used to fabricate the electrodes. Typically, 7.5 mg catalyst, 450 μL isopropanol, and 80 μL Nafion117 solution were sonicated for 20 min until a uniform slurry was obtained. Then, the slurry was coated onto a carbon gas diffusion layer (GDL; SI‐GRACET 28BC, SGL Technologies) substrate. The obtained electrode was dried at room temperature overnight in the air. The mass loading of each catalyst was ≈1.67 mg cm^−2^.

### 2.5 Electrochemical CO_2_RR Tests

The CO_2_RR experiments were performed on the Biologic potentiostat in a commercial flow cell (ElectroCell A/S) at room temperature. All tests were conducted in a chronopotentiometry (CP) mode. The cell configuration was composed of three chambers and three electrodes, as shown in Figure S1, Supporting Information. An anion exchange membrane (Sustainion X37‐50 Grade RT, Dioxide Materials) divided the cell into anodic and cathodic sides, and the cathode further divided the cathodic side into catholyte and CO_2_ gas chambers. Unless specified, both anolyte and catholyte were 1 M KOH aqueous solution. The working electrode was a catalyst‐coated GDL with a geometric area of 1.5 cm^2^. A mini‐Ag/AgCl (1 mm, leak‐free LF‐1) and IrO_2_‐coated Titanium mesh were used as reference and counter electrodes, respectively. All reported potentials were rescaled to the reversible hydrogen electrode (RHE). The details of FE calculations for gaseous and liquid products are provided in the Supporting Information.

## 3 Results and Discussion

### 3.1 Physicochemical Characterization of the As‐Prepared Catalysts

The atomic ratios of Cu to Sn in the as‐prepared bimetallic catalysts were analyzed by ICP‐OES. As shown in Table S1, Supporting Information, the actual Cu:Sn ratios are 3.1, 1.9, and 0.53 for the Cu_8_Sn_2_, Cu_7_Sn_3_, and Cu_5_Sn_5_ samples, respectively. At lower Cu:Sn ratios, the actual values closely match the nominal ones, whereas at the highest ratio, the Cu content is significantly lower than expected.

**Figure 1:**
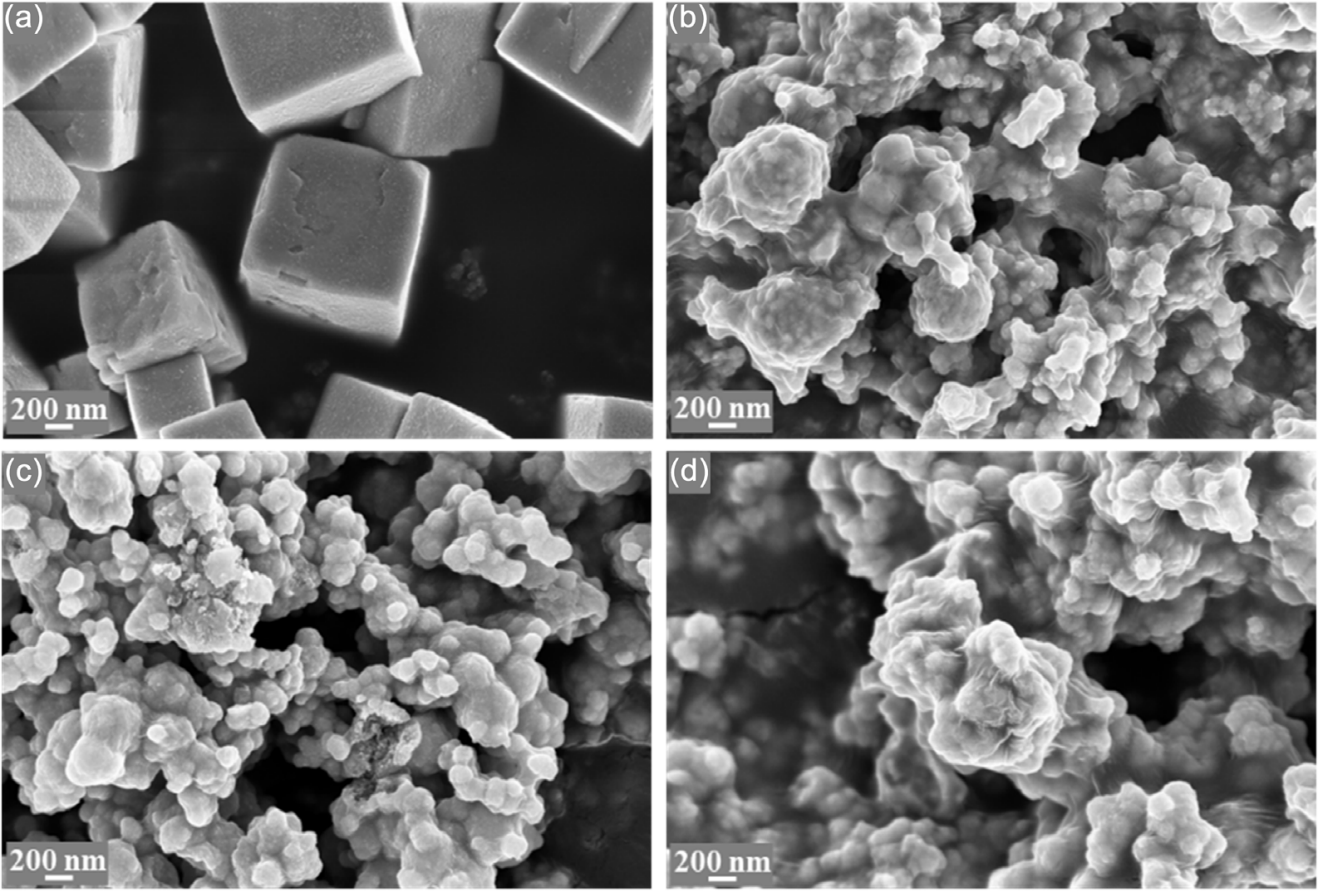
FESEM images of as‐prepared catalysts. a) Cu_2_O, b) Cu_8_Sn_2_, c) Cu_7_Sn_3_, and d) Cu_5_Sn_5_.

FESEM was employed to investigate the morphological features of the as‐prepared materials (**Figure** **1**a–d). The Cu_2_O sample consists of micrometer‐sized cubic particles. Upon the addition of Sn, a significant morphological transformation is observed, independent of the Sn concentration. All CuSn catalysts exhibit similar morphology characterized by agglomerates of densely packed nanoparticles.

**Figure 2:**
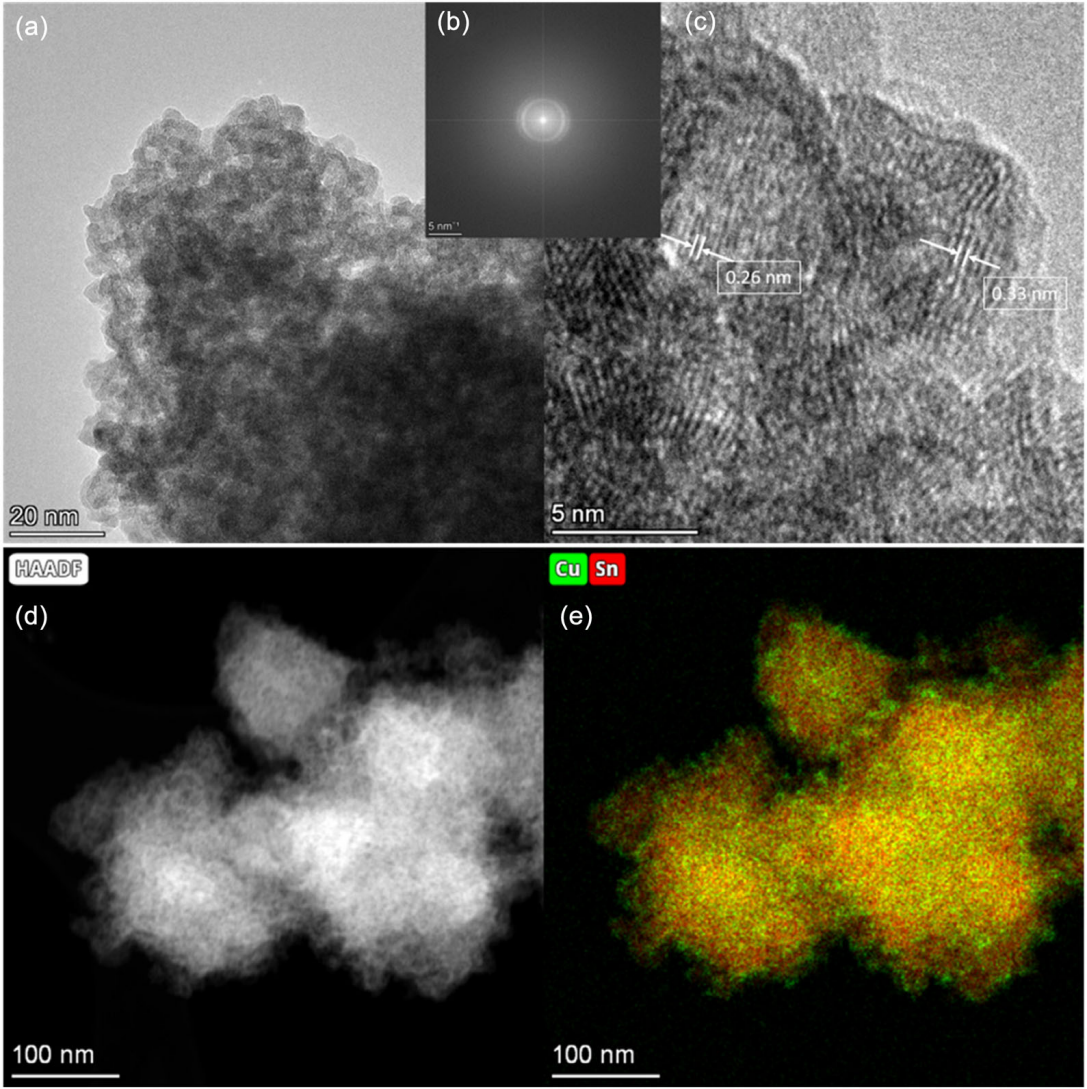
High‐magnification TEM images of the compound Cu_5_Sn_5_. a) Zoomed view displaying the average size of particles in the agglomerates, b) FFT of the image reported in (a), c) magnified view showing the two lattice spacings which were found in the structure, d) HAADF‐STEM image of and e) the relative abundance of Cu and Sn obtained by EDX analysis in the same area.

TEM analysis was performed on the selected Cu_5_Sn_5_ sample, confirming the agglomerations of very small nanoparticles with a broad distribution in size (about 2 to 7 nm in diameter). TEM images of the sample are shown in **Figure** **2**a,c. Figure 2b reports the Fast Fourier transform (FFT) of the image in Figure 2a. These images demonstrate that the nanoparticles are crystalline, displaying lattice fringes with interplanar spacings predominantly of 0.33–0.34 nm, which can be indexed to the (110) planes of SnO_2_. A minority of fringes with spacings of 0.25–0.26 nm are also observed, consistent with the (101) planes of SnO_2_.^[^ ^24^ ^]^ Figure 2d,e shows an EDX map of the sample acquired in scanning TEM (STEM mode), where the local abundance of Sn and Cu is displayed along with the high‐angle annular dark‐field (HAADF) image of the sample portion. These analyses show the presence of Cu and Sn all over the sample with a distribution not always uniform. While the main part of the agglomerations shows a homogeneous ratio of Sn and Cu, there are also a certain number of nanoparticles showing a higher fraction of Cu. Such particles are likely to be preferentially located on the outer surfaces of agglomerations.

**Figure 3:**
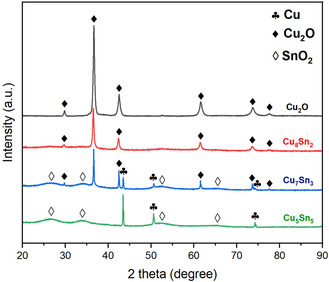
XRD patterns of synthesized catalysts.

**Figure** **3** presents the XRD patterns of all as‐prepared catalysts. The Cu_2_O sample displays diffraction peaks corresponding to the crystalline structure of cuprous oxide (Cu_2_O, PDF #01‐1142), with no detectable impurities. A similar pattern is observed for the Cu_8_Sn_2_ sample, showing no significant presence of crystalline Sn phases, as further confirmed by the higher‐resolution plotting (Figure S2, Supporting Information). Increasing the Sn content in the Cu_7_Sn_3_ sample leads to the appearance of crystalline SnO_2_ (PDF #01‐0657) and metallic Cu (PDF #01‐01241). In the Cu_5_Sn_5_ sample, which contains the highest Sn concentration, only peaks corresponding to SnO_2_ and metallic Cu are observed. The broad peaks are attributed to the small crystallite size of SnO_2_.^[^ ^24^ ^]^

**Figure 4:**
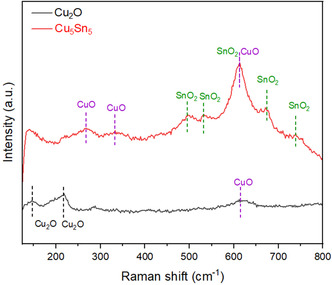
Raman spectra of synthesized Cu_2_O and selected CuSn sample.

Raman spectroscopy analysis was performed on selected samples, as displayed in **Figure** **4**. The Raman spectrum of Cu_2_O reveals its preliminary oxidation states, with a clear signature of Cu_2_O as evidenced by the most prominent peak at 217 cm^−1^ and a further peak at 145 cm^−1^. Weak signals from CuO are observed at 271 cm^−1^, 332 cm^−1^, and 611 cm^−1^.^[^ ^30^, ^31^ ^]^ The small amount of CuO is attributed to surface oxidation upon air exposure, typically forming in an amorphous state or in quantities too low to be detected by XRD.^[^ ^32^, ^33^ ^]^ Raman spectrum of Cu_5_Sn_5_ sample shows a dominant feature at 611 cm^−1^ and some additional weaker features at 499 cm^−1^, 535 cm^−1^, 675 cm^−1^, and 745 cm^−1^, which arise from the vibrations in SnO_2_ nanocrystals with a shift related to the interaction of SnO_2_ with CuO, in line with previously reported observations.^[^ ^31^, ^34^ ^]^ Both Cu_8_Sn_2_ and Cu_7_Sn_3_ catalysts show a surface composed of Cu oxides and SnO_2_ (Figure S3, Supporting Information).

In summary, the CuSn samples exhibit similar morphologies to one another, but show notable differences compared to the Cu_2_O sample. In contrast, their chemical composition is strongly influenced by the Cu‐to‐Sn ratio: increasing Sn content promotes the formation of SnO_2_ and metallic Cu. Nonetheless, Cu and Sn are homogeneously distributed at the nanoscale, with only a slight enrichment of Cu at the edges.

### 3.2 Electrochemical Characterization

**Figure 5:**
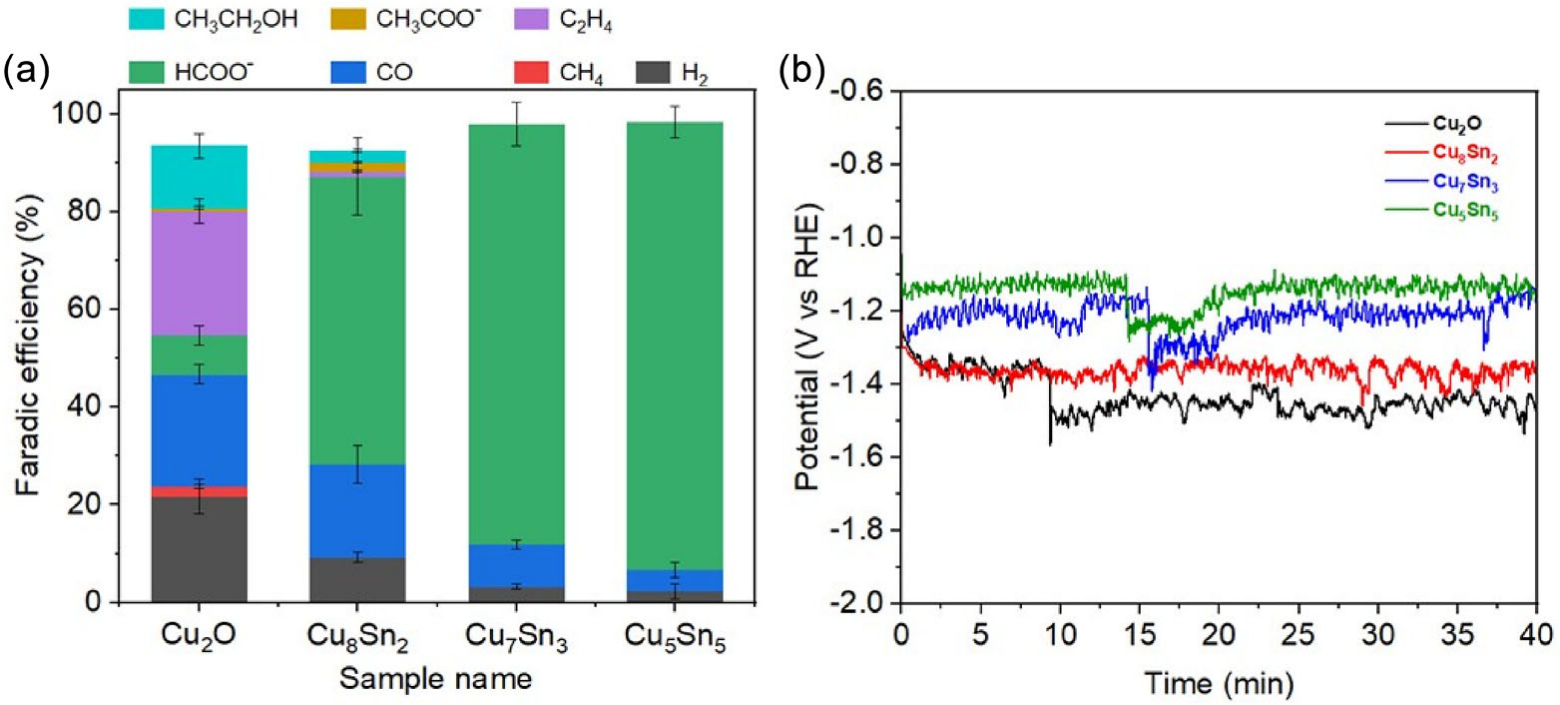
CO_2_RR performance of various electrodes at −200 mA cm^−2^ in 1 M KOH. a) FEs of different products and b) corresponding chronopotentiometry curves.

The catalytic performance toward the CO_2_ RR of various CuSn electrodes was explored in 1 M KOH alkaline solution at a current density of −200 mA cm^−2^. The Cu_2_O electrode was also tested for comparison. As shown in **Figure** **5**a, the Cu_2_O electrode mainly produces CO and C_2_H_4_, with a scarce selectivity of 6% for formate. In contrast, all CuSn electrodes exhibit a significantly enhanced selectivity toward formate production. The Cu_8_Sn_2_ sample shows a FE_HCOO–_ of 55%, which further increases to 86% and 92% for Cu_7_Sn_3_ and Cu_5_Sn_5_, respectively. It is interesting to note that the potential follows a clear trend at the applied current density: Cu_5_Sn_5_ > Cu_7_Sn_3_ > Cu_8_Sn_2_ > Cu_2_O, reflecting the same order in the catalytic activity of these electrodes. These results indicate that the addition of Sn plays a crucial role in shifting the selectivity of Cu toward formate production, and its concentration significantly influences the enhancement of formate formation. The enhanced performance observed in high Sn‐loading samples could be attributed to a cooperative effect between metallic Cu and SnO_2_, where metallic Cu enhances electron transfer,^[^ ^32^ ^]^ while SnO_2_‐derived sites favor formate formation.^[^ ^35^, ^36^ ^]^

**Figure 6:**
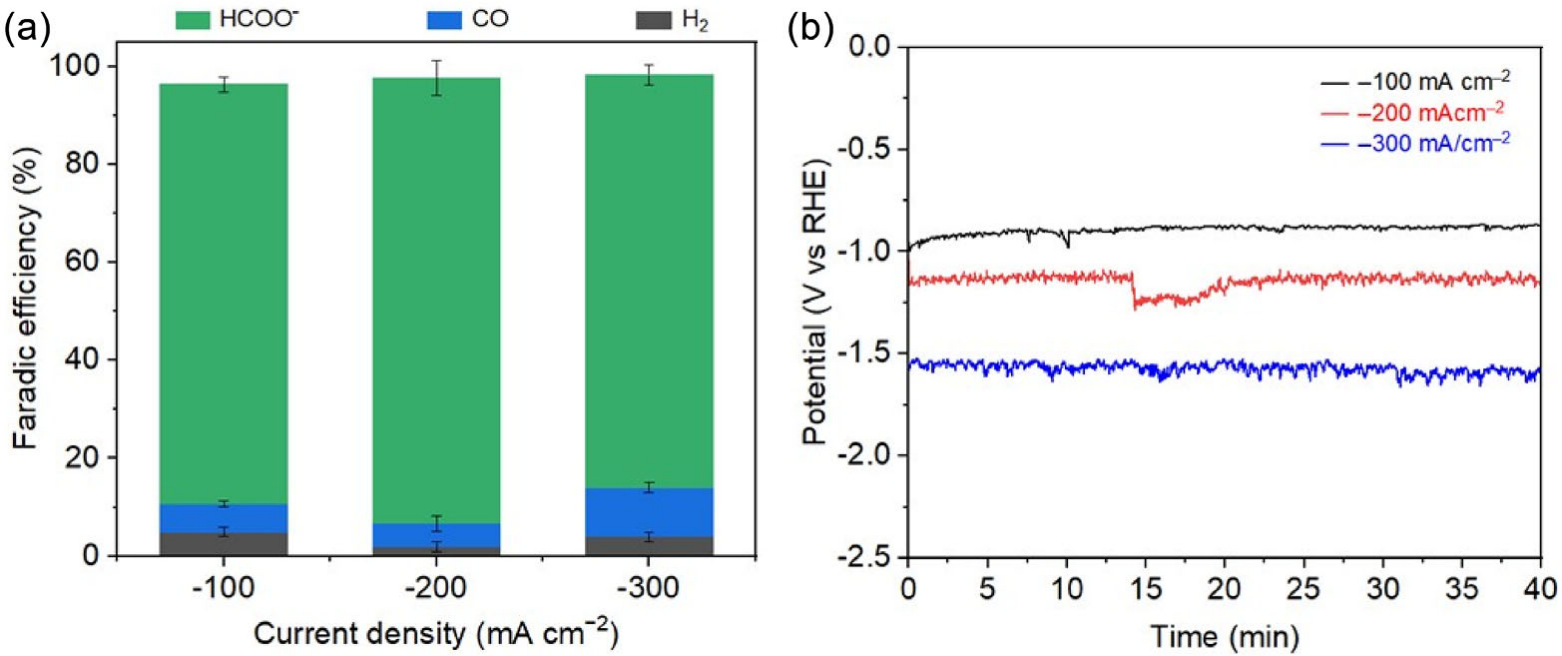
CO_2_RR performance of Cu_5_Sn_5_ catalyst at different current densities in 1 M KOH. a) FEs of various products and b) corresponding chronopotentiometry curves.

The Cu_5_Sn_5_ bimetallic catalyst was further evaluated at current densities of −100 and −300 mA cm^−2^. As shown in **Figure** **6**a, it maintains consistently high selectivity for formate across the tested current range, with faradaic efficiencies between 85% and 92%. Figure 6b shows that the potential remains nearly stable at each current density, with a gradual negative shift as the current increases, suggesting no significant mass transport limitations. The combination of high selectivity and relatively low overpotentials highlights the Cu_5_Sn_5_ catalyst as a highly promising candidate for CO_2_‐to‐formate conversion.

**Figure 7:**
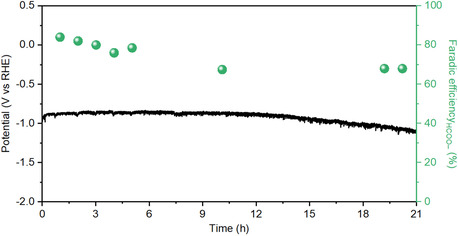
Long‐term CO_2_RR test on Cu_5_Sn_5_ electrode at −100 mA cm^−2^.

The stability of the Cu_5_Sn_5_ catalyst was assessed through a long‐term test in a flow cell using 1 M KOH electrolyte. As shown in **Figure** **7**, the FE_HCOO–_ remains above 80% during the first 5 h, followed by a gradual decline of ≈10% over the subsequent 15 h. The potential stays stable for 12 h before experiencing a gradual negative shift of about 200 mV. The decline in performance is likely due to a combination of factors and cannot be attributed to a single cause. However, since no backpressure was applied to the gas or liquid lines during the test, gradual electrode flooding over time is a plausible explanation. In any case, the observed stability is consistent with the best‐performing systems reported in the literature under similar experimental conditions.^[^ ^13^, ^37^ ^]^

**Figure 8:**
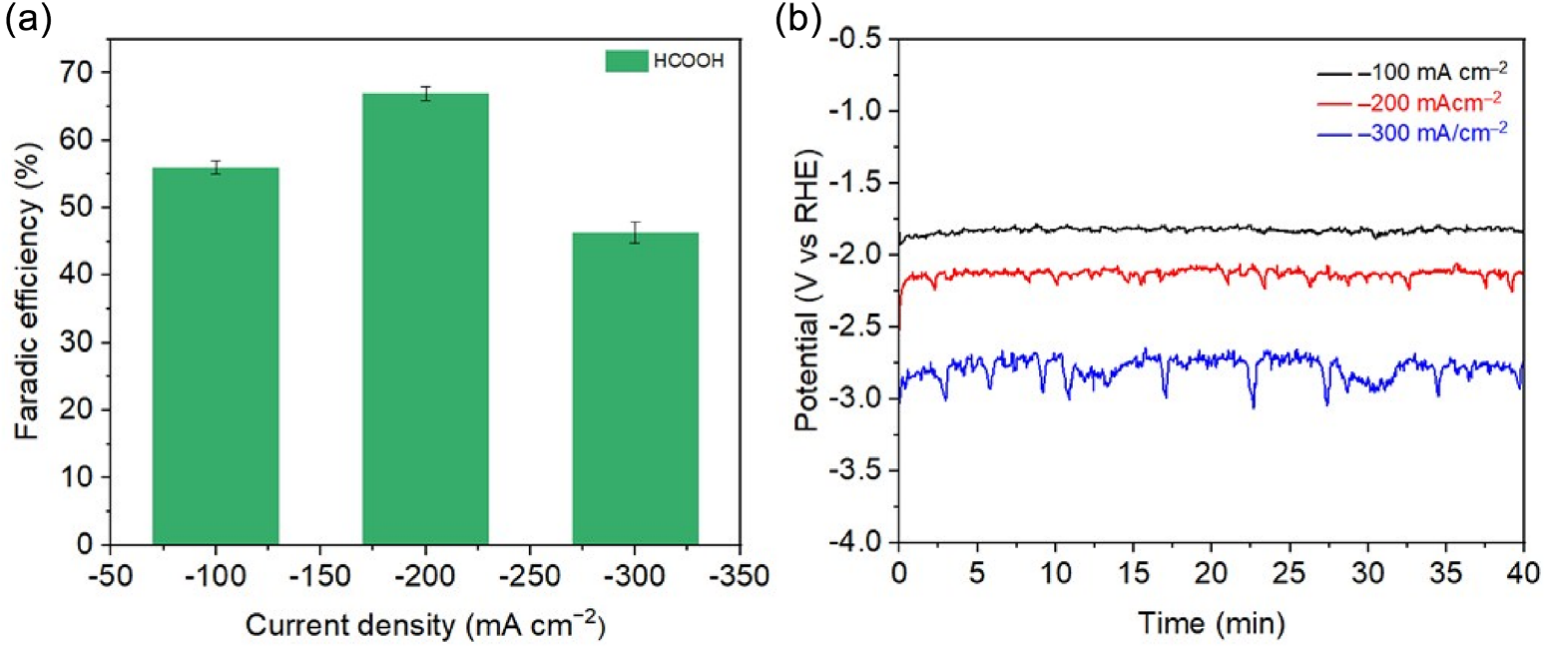
CO_2_RR performance of Cu_5_Sn_5_ catalyst in 0.5 M K_2_SO_4_ (pH 3). a) FEs of HCOOH at various current densities and b) corresponding CO_2_RR chronopotentiometry curves.

In alkaline electrolytes, CO_2_ reduction primarily yields formate rather than HCOOH, due to the high pH and the low pKa of HCOOH (3.75). As a result, an additional acidification step is required to convert formate into the more valuable HCOOH, increasing the overall process cost. Direct production of HCOOH requires acidic electrolytes (pH < 3). However, in such conditions, the hydrogen evolution reaction (HER) typically dominates due to the high proton concentration at the electrode/electrolyte interface, often suppressing CO_2_RR.^[^ ^38^, ^39^ ^]^ Thus, the catalyst's selectivity plays a more critical role under acidic conditions. To evaluate the potential of Cu_5_Sn_5_ for direct HCOOH production, CO_2_RR tests were conducted in 0.5 M K_2_SO_4_ (pH 3) using a flow cell. The acidic electrolyte was prepared by adding concentrated H_2_SO_4_ to a 0.5 M K_2_SO_4_ solution, maintaining a total K^+^ concentration of ≈1.0 M. During testing, the catholyte was circulated in single‐pass mode. As shown in **Figure** **8**, the Cu_5_Sn_5_ electrode predominantly produces HCOOH across the tested current densities, reaching a peak FE of 70% at −200 mA cm^−2^. Compared to alkaline conditions, the acidic setup requires a more negative potential to reach the same current density, indicating that the CuSn electrode exhibits higher activity in a basic environment. To understand this phenomenon, the electric double layer capacitance (*C*_dl_) of Cu_5_Sn_5_ electrodes after tests in pH 3 and 14 electrolytes was assessed. As illustrated in Figure S4, Supporting Information, the *C*_dl_ at pH 14 is 2.5 times as high as that at pH 3, highlighting a significant enhancement in the electrochemically active surface area (ECSA) under alkaline conditions. A higher ECSA provides more active sites, thereby boosting the overall electrode activity in a basic electrolyte.

### 3.3 Comparison of Synthesized CuSn Catalysts with Literature

The catalyst proposed in this study shows superior activity and selectivity for formate production in CO_2_RR compared to previously reported Cu‐ and Sn‐based systems (**Table** **1**). The Cu and Sn sources are low‐cost and abundantly available. Moreover, the microwave‐assisted one‐pot synthesis is energy‐efficient, rapid, and reproducible. These excellent performances highlight the strong potential of the proposed catalysts for practical applications.

**Table 1:** Performance comparison of various electrocatalysts for CO_2_RR to formate.

Catalyst	Electrolyte	*j*_HCOO−_ [mA cm^−2^]	Potential (V versus RHE)	FE_HCOO−_ [%]	Reference
Cu_5_Sn_5_	1 M KOH	−184	−1.1	92	This work
Cu_5_Sn_5_	0.5 M K_2_SO_4_ (pH 3)	−140	−2.1	70	This work
Cu‐SnS_2_	0.5 M KHCO_3_	−23.8	−1.0	90.5	[23]
5%Cu‐SnO_2_	0.5 M KHCO_3_	−22	−0.9	91	[24]
Cu_3_Sn/Cu_6_Sn_5_	1 M KOH	−18.9	−1.0	87	[25]
Hemicapsule Cu@SnO_2_	0.1 M KHCO_3_	−28	−1.35	67	[26]
CuSn NPs/C‐A	0.1 M KHCO_3_	−12.6	−1.0	71.5	[27]
CuS/SnO_2_‐S	0.5 M KHCO_3_	−18.8	−0.8	84.9	[28]
CuO/SnO_2_	1 M KOH	−3.19	−1.05	57.5	[40]
R‐CuSnO_3_	1 M KOH	−26	−0.9	93.4	[41]
CuO–SnO_2_ NFs	1 M KOH	−186	−0.7	74.3	[42]
CuSn HFGDE	0.5 M KHCO_3_	−68	−1.2	78	[43]
Cu–Sn aerogel	0.5 M KHCO_3_	−21.8	−1.1	90.8	[44]

## 4 Conclusion

This study presents CuSn bimetallic catalysts synthesized via a simple microwave‐assisted method for the efficient electrochemical conversion of CO_2_ to formate/formic acid. The catalyst morphology remains largely unchanged across different Sn contents, from 25 at% to 65 at%. However, the phase composition varies significantly with Sn content: the low‐Sn sample predominantly exhibits crystalline Cu_2_O, whereas the high‐Sn sample shows crystalline phases of metallic Cu and SnO_2_. These compositional differences strongly influence the selectivity of the CO_2_ reduction reaction, highlighting the critical role of Sn content in tuning catalyst performance. Specifically, the high‐Sn sample demonstrates excellent selectivity (92%) and good activity (−1.1 V versus RHE at −200 mA cm^−2^) for formate production in alkaline conditions. This remarkable performance could be attributed to the synergistic interaction between metallic Cu and SnO_2_, where the former facilitates electron transfer and the SnO_2_‐derived sites promote formate formation. Moreover, it maintains a promising selectivity of 70% for direct formic acid production in acidic electrolyte. This study offers a new pathway for developing high‐performance and low‐cost catalysts for industrial‐scale CO_2_ conversion into valuable chemical products.

## Conflict of Interest

The authors declare no conflict of interest.

## Supporting information

Supplementary Material

## Data Availability

The data that support the findings of this study are available from the corresponding author upon reasonable request.
