# Supplementary material for: Nanoscale Copper–Tin Dioxide Interfaces for Efficient CO2 Electroreduction to Formic Acid and Formate at High Rates
Source: ChemSusChem. 2025 Nov 3;18(24):e202501686. doi: 10.1002/cssc.202501686 (PMC12703421; doi:10.1002/cssc.202501686)
Supplement: Supplementary file 1 — Supplementary Material [file CSSC-18-e202501686-s001.pdf]

Support Information

Nanoscale Copper–Tin Dioxide Interfaces for Efficient CO_2 Electroreduction to Formic Acid and Formate at High Rates

Lan Huang[1,2], Felicia Di Costola[1,2], Marco Allione[2], Stefano Bianco[2], Adriano Sacco[1], Candido F. Pirri[1,2], Juqin Zeng[1,2*]*

1. Istituto Italiano di Tecnologia – IIT, Centre for Sustainable Future Technologies (CSFT), Via Livorno 60, Turin, 10144, Italy
2. Department of Applied Science and Technology (DISAT), Politecnico di Torino, Corso Duca degli Abruzzi 24, Turin,10129, Italy

Products Analysis

Gas chromatograph (μGC, Fusion, INFICON) with a 10 m Rt-Molsieve 5A column and an 8 m Rt-Q-Bond column was used to detect gas products. The FE calculation for gaseous products[1] is:

$$FE_{gas} = V*t*C*n*F / V_m*Q$$

where the V_m represents the molar volume of an ideal gas (L mol^{-1}), and V indicates the flow rate of CO_2 at the cathodic side (L min^{-1}), the electrolysis duration time (min) is used by t, Q is the total charge passed through the system during the electrolysis time t (coulombs, C), C is the concentration of the gas product (% v/v), n is the number of electrons required to obtain one molecule of this product (n = 2 for CO and H_2 formation), and F is the Faraday constant (96 485 C mol^{-1}).

In addition, liquid products were quantified by a high-performance liquid chromatograph (Shimadzu Prominence HPLC) with a Diode Array Detector (DAD) set at 210 nm by using a Rezex ROA (300 × 7.8 mm) column, with 5 mM H_2SO_4 (flow rate of 0.5 mL min^{-1}) as mobile phase. The FE of $HCOO^-$ can be calculated as follows：

$$FE = 2FVC / Q \times 100\%$$

F is faradaic constant, V is the volume of electrolyte in cathode compartment, C is the concentration of formate or formic acid in electrolyte and Q is the charge passed through catalyst.

Double-layer capacitance (C_{dl}) measurements

The electric double-layer capacitance (C_{dl}) of GDEs was estimated by performing CV scans in 0.5 M K_2SO_4 and 1 M KOH at scan rates from 20 to 100 mV s^{-1} over a 0.1 V electrochemical window where no Faradaic reaction happens.

The double layer can be calculated via the following equation[2]:

$$C_{dl}= j/(dV/dt)$$

where C is the capacitance, j is the current density at the center of the -0.15 V vs RHE electrochemical window, and dV/dt is the CV scan rate. The scan rates are 20, 40, 60, 80 and 100 mV/s, respectively.

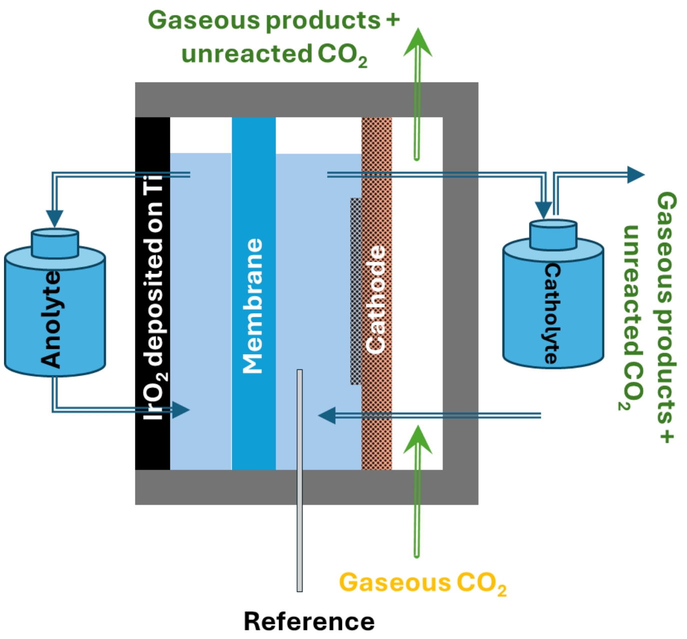

Figure S1. The illustration of flow cell for CO_2RR.

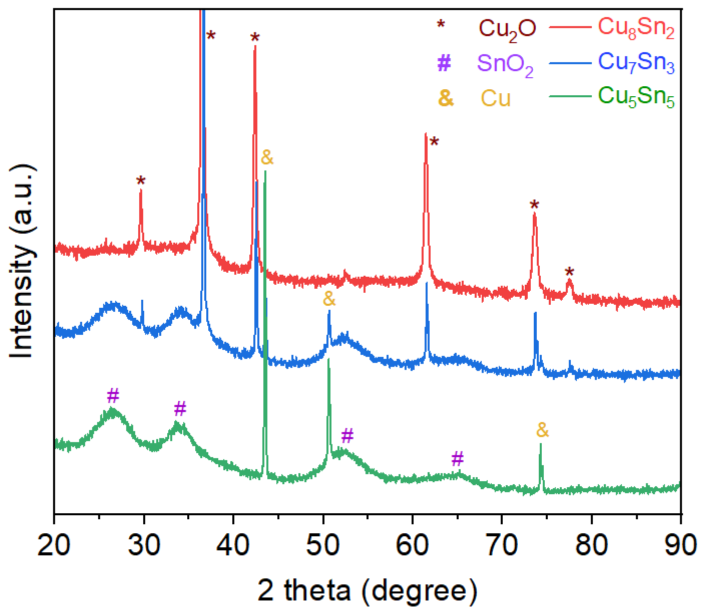

Figure S2. XRD patterns of CuSn samples at a higher resolution.

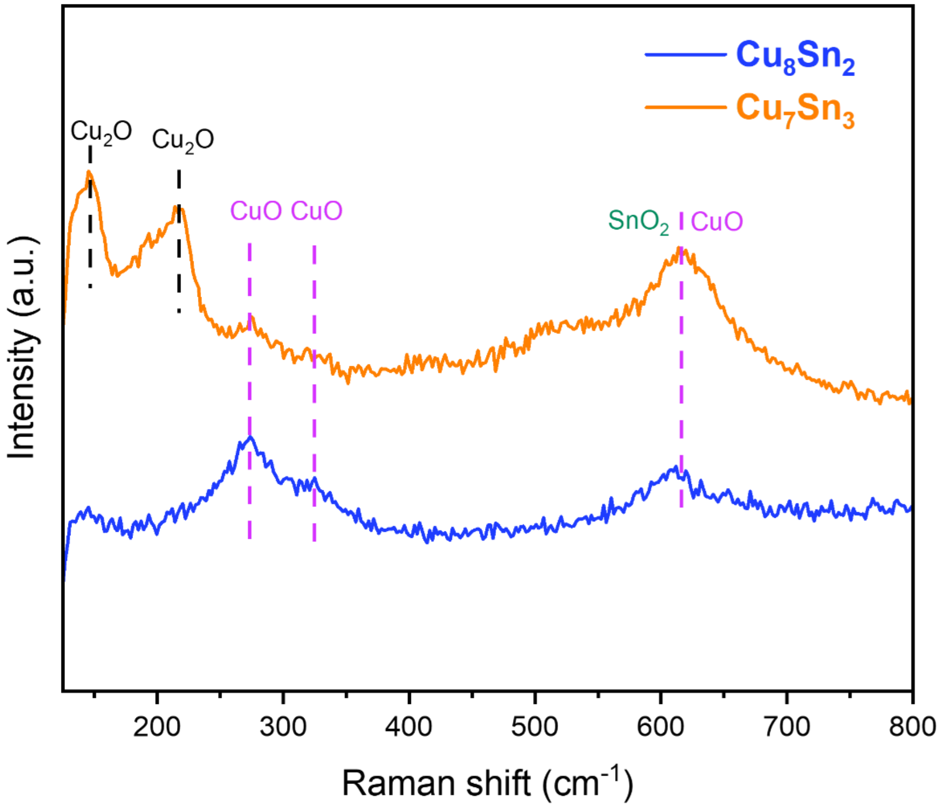

Figure S3. Raman spectroscopy of Cu_8Sn_2 and Cu_7Sn_3 samples.

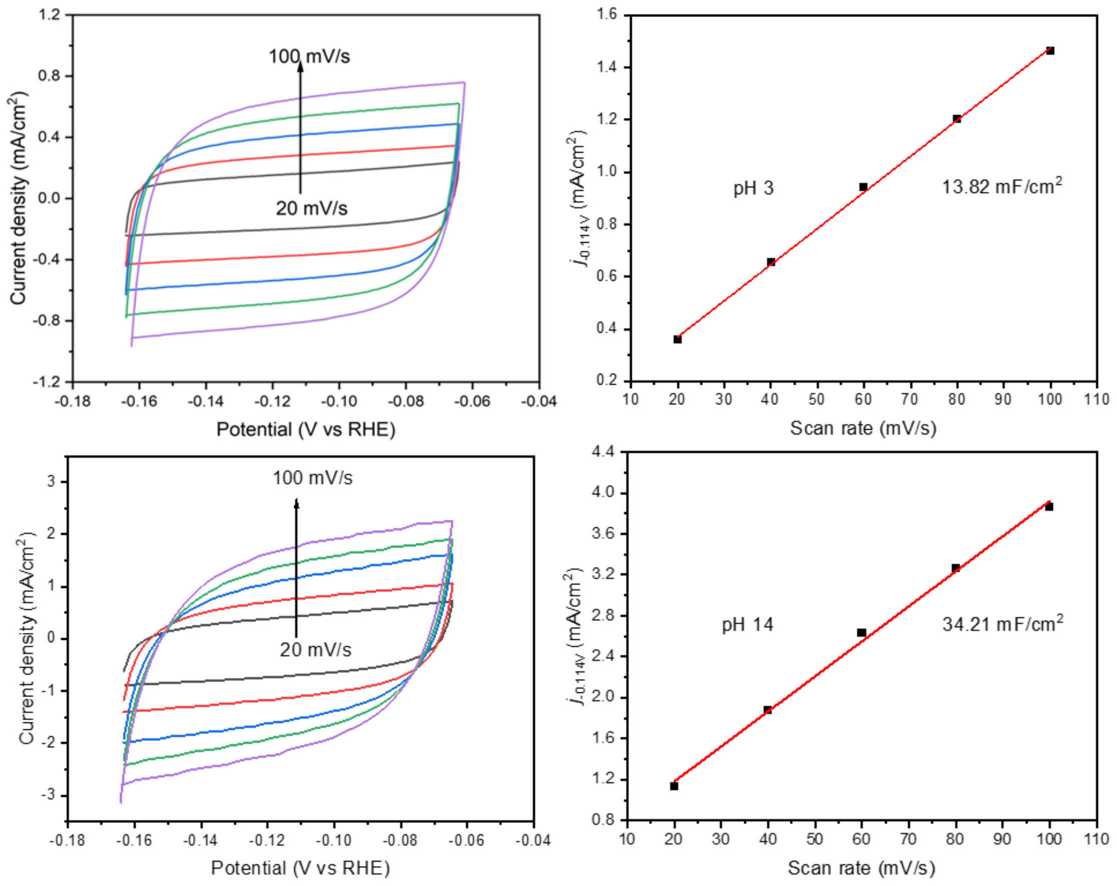

Figure S4. C_{dl} of catalyst Cu_5Sn_5 after tests in electrolytes with pH 3 and 14, respectively.

Table S1. Atomic percentages of Cu and Sn in the prepared catalysts, as defined by ICP.

**Sample**	**Element**	**Percentage (at.%)**
Cu_2O	Cu	100%
Cu_8Sn_2	Cu	75%
	Sn	25%
Cu_7Sn_3	Cu	65%
	Sn	35%
Cu_5Sn_5	Cu	35%
	Sn	65%

Reference:

[1] Monti, N. B. D.; Fontana, M.; Sacco, A.; Chiodoni, A.; Lamberti, A.; Pirri, C. F.; Zeng, J. Facile Fabrication of Ag Electrodes for CO2-to-CO Conversion with Near-Unity Selectivity and High Mass Activity. *ACS Appl. Energy Mater.* **2022**, *5* (12), 14779–14788. https://doi.org/10.1021/acsaem.2c02143.

[2] Rabiee, H.; Zhang, X.; Ge, L.; Hu, S.; Li, M.; Smart, S.; Zhu, Z.; Yuan, Z. Tuning the Product Selectivity of the Cu Hollow Fiber Gas Diffusion Electrode for Efficient CO2 Reduction to Formate by Controlled Surface Sn Electrodeposition. *ACS Appl. Mater. Interfaces* **2020**, *12* (19), 21670–21681. https://doi.org/10.1021/acsami.0c03681.
